# Combination of Sorafenib and Transarterial Chemoembolization in Selected Patients with Advanced-Stage Hepatocellular Carcinoma: A Retrospective Cohort Study at Three German Liver Centers

**DOI:** 10.3390/cancers13092121

**Published:** 2021-04-28

**Authors:** Christine Koch, Markus Göller, Eckart Schott, Oliver Waidmann, Mark op den Winkel, Philipp Paprottka, Stephan Zangos, Thomas Vogl, Wolf Otto Bechstein, Stefan Zeuzem, Frank T. Kolligs, Jörg Trojan

**Affiliations:** 1 Department of Medicine 1, University Hospital Frankfurt, Goethe-University, Theodor-Stern-Kai 7, 60590 Frankfurt, Germany; christine.koch@kgu.de (C.K.); markus.goeller@uk-erlangen.de (M.G.); oliver.waidmann@kgu.de (O.W.); zeuzem@em.uni-frankfurt.de (S.Z.); 2 University Cancer Centre, University Hospital Frankfurt, Goethe-University, 60590 Frankfurt, Germany; 3 Department of Gastroenterology, Hepatology and Diabetology, Internal Medicine II, HELIOS Hospital Emil von Behring, 14165 Berlin, Germany; eckart.schott@helios-gesundheit.de; 4 Department of Medicine II, University Hospital, Ludwig Maximilians University, 81377 Munich, Germany; Mark.op.den.Winkel@med.uni-muenchen.de (M.o.d.W.); philipp.paprottka@mri.tum.de (P.P.); frank.kolligs@helios-gesundheit.de (F.T.K.); 5 Department of Interventional Radiology, Klinikum rechts der Isar, Technical University Munich, 80333 Munich, Germany; 6 Department of Diagnostic and Interventional Radiology, University Hospital Frankfurt, Goethe-University, 60590 Frankfurt, Germany; stephan.zangos@af-k.de (S.Z.); thomas.vogl@kgu.de (T.V.); 7 Department of Radiology and Nuclear Medicine, Klinik am Eichert und Helfenstein Klinik, 73312 Göppingen, Germany; 8 Department of General and Viszeral Surgery, University Hospital Frankfurt, Goethe-University, 60590 Frankfurt, Germany; wolf.bechstein@kgu.de; 9 Department of Internal Medicine and Gastroenterology, Helios-Klinikum Berlin-Buch, 13125 Berlin, Germany

**Keywords:** HCC, TACE, sorafenib, treatment

## Abstract

**Simple Summary:**

Transarterial chemoembolization (TACE) is the treatment of choice for patients with liver cancer without distant metastases or tumor growth into blood vessels. For the latter patients, sorafenib is a well-established oral medication. Combination of both treatments might also enhance effectiveness and survival in patients with advanced tumor stages. We retrospectively compared patients with advanced liver cancer (with distant metastases and/or tumor growth into blood vessels) from three German university hospitals who received either TACE alone, sorafenib alone or the combination treatment. We found that survival was prolonged for patients receiving the combination treatment without increasing frequency or severity of side effects. These results are in line with published results from Asian patients and show that this treatment might also be feasible in a Western population for selected patients with advanced liver cancer.

**Abstract:**

Background and Aims. Systemic treatment with sorafenib has been the standard of care (SOC) in patients with advanced Barcelona Clinic Liver Cancer (BCLC) stage C hepatocellular carcinoma (HCC) for more than a decade. TACE has been reported to allow better local tumor control in selected patients with BCLC stage C HCC. Methods. A retrospective analysis of patients with BCLC stage C HCC that were treated with sorafenib and TACE was conducted; they were compared to BCLC stage C patients treated either with TACE or sorafenib in the same period of time outside a clinical trial. Results. A total of 201 patients with BCLC stage C were identified, who were treated with either sorafenib and TACE (group A; *n* = 54), sorafenib (group B; *n* = 82) or TACE (group C; *n* = 65). No significant difference in baseline characteristics was observed. Time to progression was 7.0 months (95% CI: 4.3–9.7), 4.1 months (95% CI: 3.6–4.7) and 5.0 months (95% CI: 2.9–7.1) in groups A, B and C, respectively, and overall survival was 16.5 months (95% CI: 15.0–18.1), 8.4 months (95% CI: 6.0–10.8) and 10.5 months (95% CI: 7.5–13.6), respectively (group A vs. group B: *p* < 0.001; group A vs. group C: *p* = 0.0023). Adverse events of grade 3/4 occurred in 34% of patients in group A. Conclusions. Although sorafenib is a SOC in patients with BCLC stage C HCC, TACE is frequently used as an additional locoregional treatment in selected patients. This combined approach resulted in a significant overall survival benefit in selected patients, although randomized trials have not yet proven this benefit.

## 1. Introduction

The worldwide incidence of hepatocellular carcinoma (HCC) is rising, with an annual incidence of above 600,000 patients [1]. Treatment of HCC is challenging because HCC mainly occurs within liver cirrhosis, and therapeutic options and prognosis are determined by tumor biology as well as impaired liver function [2]. Currently, the most commonly used clinical staging system in Western countries is the Barcelona Clinic Liver Cancer (BCLC) algorithm [3,4]. According to BCLC, treatment is stratified depending on tumor stage, liver function and performance status. Intermediate-stage HCC (BCLC stage B) without options for surgical treatment or ablation is best treated by transarterial chemoembolization (TACE), which has been shown to extend median survival from 16 to 20 months [5,6]. Response rates after TACE treatment are in the range of about 35% [6,7]. Advanced-stage HCC (BCLC stage C) is defined by portal vein infiltration (PVI), extrahepatic tumor manifestation (EHM) and/or a reduced Eastern Cooperative Group (ECOG) performance status. In patients with BCLC stage C, treatment with sorafenib, an oral multi-tyrosine kinase and angiogenesis inhibitor with activity against vascular endothelial growth factor receptor (VEGFR)-2, PDGFR, c-Kit receptors, BRAF and p38 signal transduction pathways, was considered the standard of care (SOC) at the time the study was conducted. Two independent pivotal phase 3 trials demonstrated a survival benefit compared to a placebo in Caucasian and Asian patients with HCC [8,9]. Prognosis in advanced-stage HCC is strongly dependent on the preservation of liver function, and the majority of patients with BCLC stage C die because of either liver failure or intrahepatic progression [10,11]. Since TACE is also feasible in patients with side-branch PVI, some investigators achieved overall survival rates comparable to treatment with sorafenib in selected patients with HCC BCLC stage C [12,13]. Treatment with TACE leads to vascular endothelial growth factor (VEGF) upregulation in HCC patients [14]. Since sorafenib also targets VEGF [15], a combination of sorafenib as an inhibitory factor with TACE might decrease neovascularization and, therefore, potentiate the effect of chemoembolization, though this has never been shown in a randomized trial in BCLC stage B patients thus far for several possible reasons, including the high technical variability between different liver centers. However, two reports from China suggest that the combination of sorafenib and TACE in advanced HCC is also feasible, and the efficacy is encouraging [16,17]. To compare the efficacy of the combination of sorafenib and TACE to either TACE alone or sorafenib alone in Western HCC patients, a retrospective cohort study was initiated at three German liver centers.

## 2. Patients and Methods

Between January 2007 and December 2012, a consecutive cohort of HCC patients treated with sorafenib in combination with TACE (group A) at three German liver centers (Goethe-University, Frankfurt; Charité, Berlin and Ludwig Maximilians University, Munich) was studied retrospectively. Additionally, patients with advanced-stage HCC treated with either sorafenib (group B) or TACE (group C) in the same period of time were included. HCC was diagnosed according to the criteria published by the European Association for the Study of Liver Disease/American Association for the Study of Liver Disease [5]. Inclusion criteria for the study population were as follows: ECOG performance status ≤ 2, Child–Pugh class A or B (scores ≤ 8) and BCLC stage C. Only patients treated with sorafenib for a minimum of 30 days were considered. Other, e.g., loco-regional, treatments or resection in the medical history were allowed. Patients with progression from BCLC stage B to stage C while receiving TACE treatment were excluded. Complete in- and exclusion criteria are listed in Supplementary File S1. In group A, TACE was usually initiated before sorafenib. The applied conventional lipiodol-based TACE protocols at the three centers used either mitomycin C, epirubicin or doxorubicin as chemotherapeutic agents and were repeated every 4–8 weeks and terminated in case of either progression, toxicity or complete tumor devascularization. After the start of sorafenib treatment, patients were initially closely followed for 7–14 days and every 4–8 weeks thereafter, as appropriate. Intervals between radiological examinations were usually ten to twelve weeks. Overall survival and time to radiological progression were analyzed at all three centers, whereas adverse events were analyzed in detail in Frankfurt only. The ethics committee at the Goethe-University Hospital Frankfurt, Germany, approved the study.

## 3. Statistical Analyses

Statistical analyses were carried out using BiAs 10.03 software (Frankfurt, Germany) and Microsoft Office Excel 2007 (Microsoft Corporation, Redmont, WA, USA).

Patients with CLIP score >4 points and Child–Pugh score >8 were excluded from the analysis. In total, 201 patients were included in the final analysis. Continuous parameters were analyzed with descriptive methods (mean with standard deviation); the Kruskal–Wallis test was used for comparison of different groups. Categorical parameters were expressed as frequency and percentages and analyzed using Fisher’s exact test. Survival was calculated from the date of first TACE treatment or the day of first sorafenib dose until data closure, loss to follow-up or death, whichever came first. Median survival times and median times to radiological progression were analyzed with the Kaplan–Meier method and log-rank test; *p*-values < 0.05 were considered statistically significant. Uni- and multivariate analyses were carried out to identify prognostic predictors for survival time using Cox regression and log-rank tests, *p*-values < 0.05 were considered statistically significant.

## 4. Results

### 4.1. Patients

**Figure 1:**
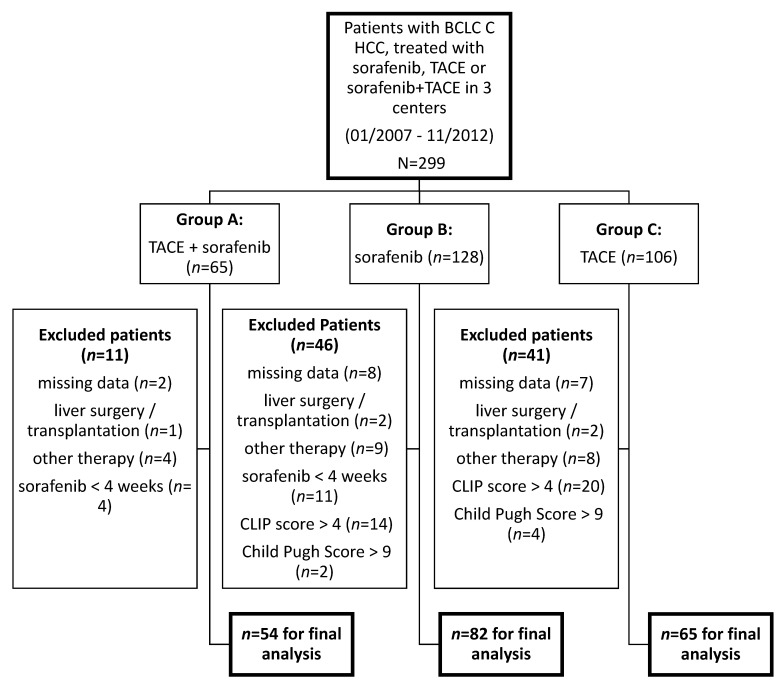
Patient characteristics.

In total, 54, 82 and 65 patients were included in groups A–C, respectively (Figure 1). There was no significant difference in baseline characteristics between the three groups (Table 1).

### 4.2. Radiologic Response

**Figure 2:**
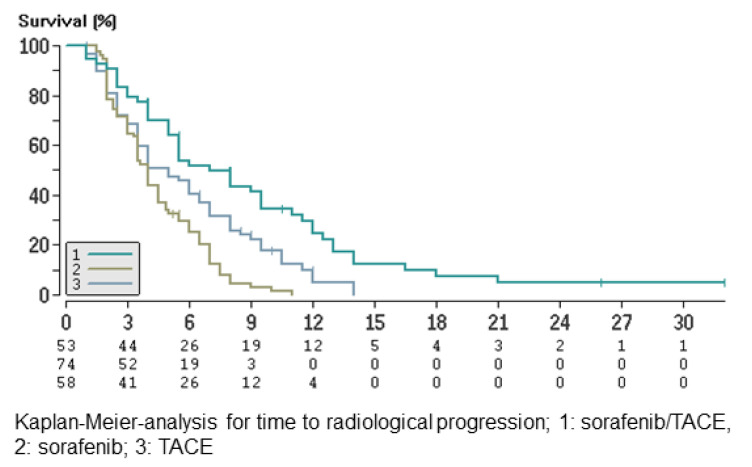
Time to radiological progression.

Based on the RECIST 1.1 tumor evaluation, the median time to radiological progression (TTP) was 7.0 months (95% CI: 4.3–9.7), 4.1 months (95% CI: 3.6–4.7) and 5.0 months (95% CI: 2.9–7.1) in groups A, B and C, respectively (Figure 2). The TTP in patients in the combination arm was significantly longer than that in groups B and C (group A vs. group B: *p* < 0.001; group A vs. group C: *p* < 0.001).

Radiological evaluation after six months showed significantly higher disease control and objective response rates in the combination group as compared to the single-treatment groups: the DCR was 53% in the sorafenib/TACE group as compared to 23% in the sorafenib group and 38% in the TACE group (*p* = 0.0024); the ORR was 15% in the combination group, 3% in the sorafenib group and 9% in the TACE group (*p* = 0.019) (Table 2).

### 4.3. Survival Times

**Figure 3:**
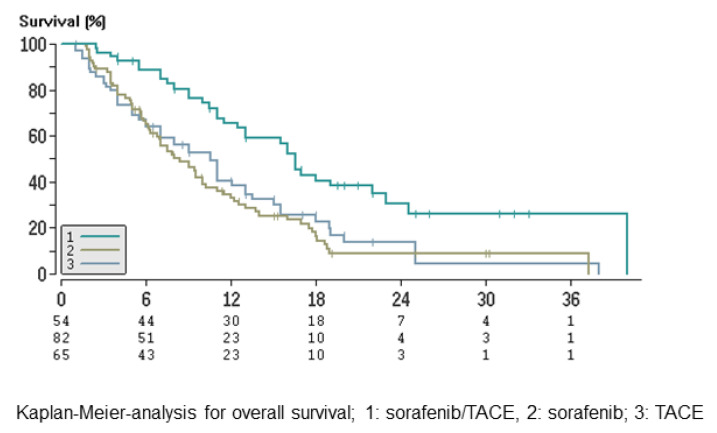
Overall survival.

Median overall survival (OS) was 16.5 months (95% CI: 15.0–18.1), 8.4 months (95% CI: 6.0–10.8) and 10.5 months (95% CI: 7.5–13.6) in groups A, B and C, respectively (Figure 3, Table 3). The OS in patients in the combination arm was significantly longer than that in groups B and C (group A vs. group B: *p* < 0.001; group A vs. group C: *p* = 0.0023). Until the end of follow-up (November 2012), 61% of patients in the sorafenib and TACE group, 81% of patients in the sorafenib group and 79% of patients in the TACE group had died. There was no significant difference between patients with or without distant metastases in the individual groups (group A: *p* = 0.59; group B: *p* = 0.27; group C: *p* = 0.99).

In the multivariate analysis, a baseline Child–Pugh score B (HR 0.47), ECOG status ≥ 1 (HR 0.56) and alpha-fetoprotein level >400 ng/dL (HR 0.56) were negative predictors of survival (Table 4).

Sorafenib-related adverse events were comparable in patients in the sorafenib/TACE group as compared to patients who received only sorafenib treatment (Table 5). A dose reduction of sorafenib was documented in 66% of patients in the combination group and in 60% in the sorafenib group. Adverse events in patients in the TACE group are listed in Table 6 and were also not significantly different between the TACE-only and combination treatment patients.

## 5. Discussion

In this multi-center, retrospective cohort study, we evaluated the efficacy and safety of sorafenib in combination with TACE in patients with advanced HCC.

We found that the combination treatment significantly prolonged the time to radiological progression, disease control and overall response rate as well as the median overall survival. Adverse events did not occur more frequently in these patients when compared to the patients receiving sorafenib or TACE only. Since all groups were well balanced, those differences cannot be attributed to differences in liver function or tumor burden. Nevertheless, due to the retrospective nature of the study, a selection bias cannot be excluded. Additionally, the technical aspect is important for interpretation of our data and the reports in the literature: TACE protocols and the technical performance of the interventional radiologist naturally differ between departments. Therefore, small groups of highly selected patients might show a benefit that probably cannot be generalized. We also did not analyze salvage treatments separately, although they might have influenced survival times.

It has been suggested that only patients with tumors without vascular invasion or extrahepatic spread (BCLC stage B) benefit from TACE [3]. However, in everyday practice, TACE is also applied in selected patients with advanced HCC to enhance local tumor control [18,19] and also in patients with grade 1 or 2 thromboses of the portal vein [20].

Clinical studies investigating the prognostic role of extrahepatic spread in HCC patients that were treated with sorafenib returned conflicting results. However, in a recent trial by Schütte et al., prognosis of HCC patients treated with a combination of SIRT and sorafenib depended mainly on the extent of liver involvement [21]. In our trial, patients with or without extrahepatic metastases did not show significant differences in their median survival. In our opinion, this justifies the selection of the criterion of BCLC stage over extrahepatic spread or different subgroups according to tumor size.

The SPACE trial, studying the effect of sorafenib in combination with doxorubicin-eluting beads and TACE in intermediate-stage HCC (BCLC stage B), showed no relevant benefit with the addition of sorafenib [22]. In our cohort with only BCLC stage C patients, every center used their preferred TACE regimen, which was, in the majority of procedures, a canonical, lipiodol-based TACE. Moreover, sorafenib dose adjustments were frequently documented. Of note, sorafenib-related adverse events were comparable in patients treated with sorafenib in combination with TACE compared to sorafenib alone. Liu et al. retrospectively compared patients with BCLC stage C HCC that received TACE and sorafenib vs. patients treated with TACE alone. They confirmed our results of a better local tumor control and longer survival for the combination group. The median OS and survival rate of the TACE monotherapy group at one year in our trial were slightly higher than those reported by Liu et al. Lee et al. analyzed patients receiving either TACE alone or in combination with sorafenib, and the survival times in the monotherapy group were shorter than those in our cohort. These differences have to be interpreted with caution, however, due to the high clinical variability of BCLC stage C patients [23,24]. Varghese et al. also analyzed a mixed dataset of BCLC stage B and C patients and found a survival benefit of the combination treatment at both stages [25]. Additionally, a recent prospective trial by Kudo et al. (TACTICS) stressed the effect of a combination treatment on local tumor control, although only a minority of patients suffered from BCLC stage C HCC [26]. Taken together, the combination treatment with TACE and sorafenib seems to be feasible and safe in BCLC stage C patients. Zhao et al. proposed [17] a risk score to further divide BCLC stage C patients into subgroups according to vascular invasion, Child–Pugh stage and tumor burden. Patients with a score of less than 11.5 should receive the combination treatment. Our trial adds value to the current knowledge by proving the treatment’s safety and feasibility in Western patients.

The patients in our study were included retrospectively and according to in- and exclusion criteria as mentioned above. Therefore, there were no major differences over the course of six years. Until the approval of lenvatinib as additional first line treatment in HCC patients in 2018, sorafenib was the only systemic treatment in HCC patients. No other (further-line) options, such as cabozantinib, regorafenib and ramucirumab as well as atezolizumab/bevacizumab, were available at the time the study was conducted and, therefore, did not influence survival analyses.

In 2018, the tyrosine kinase inhibitor lenvatinib was approved by the FDA and the EMA as an alternative first-line treatment for patients with advanced HCC, following the results of a phase III non-inferiority trial that compared sorafenib and lenvatinib in this indication [27]. One recent retrospective study investigated the combination treatment of TACE and lenvatinib in a Japanese cohort and found a survival benefit for patients when treated alternatingly [28]. The most recent development in HCC treatment was the approval of atezolizumab and bevacizumab in patients with advanced HCC in 2020 after the results of the pivotal IMbrave150 trial, which is considered as the new SOC in advanced HCC [29]. Nevertheless, the role of combined locoregional therapy and systemic therapy is being further studied, also in combination with immunotherapy NCT04803994, NCT04340193) [30]. Since advanced HCC is a very heterogenous disease as defined by BCLC and other staging systems, it is challenging to run international multi-center studies using a combination of locoregional therapy together with systemic therapy.

## 6. Conclusions

In conclusion, the results of our retrospective cohort study at three Western HCC centers indicate that carefully selected Western HCC patients might also benefit from a combination approach using TACE and the TKI sorafenib, although randomized trials have not yet proven this.

## Figures and Tables

**Table 1:** Patient characteristics.

Patients’ Characteristics	Sorafenib/TACE (Group A) *n* = 54	Sorafenib (Group B) *n* = 82	TACE (Group C) *n* = 65	*p*-Value
Median age (range)	64.0 (34–77)	65.9 (28–85)	67.0 (41–80)	0.13
Male sex	87%	88%	82%	0.56
Alcohol abuse	31%	28%	38%	0.41
Viral hepatitis	43%	35%	34%	0.56
NASH	7%	6%	8%	0.88
Cryptogenic/other causes	19%	31%	20%	0.21
BCLC stage C	100%	100%	100%	1.0
Child–Pugh A	74%	74%	77%	0.94
Child–Pugh B	26%	26%	23%	
ECOG PS ≥ 1	70%	55%	62%	0.19
Tumor burden ≥50% liver involvement	15%	22%	15%	0.86
Portal vein infiltration (grade 1–3)	33%	33%	38%	0.77
Extrahepatic spread	41%	46%	34%	0.31
α-Fetoprotein ≥400 ng/dL	34%	36%	38%	0.89
CLIP score (range)	2.1 ± 1.0 (1.0–4.0)	2.1 ± 0.8 (1.0–4.0)	2.1 ± 0.8 (1.0–4.0)	

**Table 2:** Summary of outcomes according to RECIST 1.1 evaluation after 6 months.

6-Month Radiologic Evaluation (RECIST 1.1)	Sorafenib/TACE (*n* = 53) *	Sorafenib (*n*= 74) *	TACE (*n*= 58) *	*p*-Value
Complete response	0 (0%)	0 (0%)	0 (0%)	1.0
Partial response	8 (15%)	2 (3%)	5 (9%)	0.0187
Stable disease	20 (38%)	15 (20%)	17 (29%)	0.1423
Progression of disease	25 (47%)	57 (77%)	36 (62%)	0.0024
Disease control rate ^±^	28 (53%)	17 (23%)	22 (38%)	0.0024
Objective response rate ^¶^	8 (15%)	2 (3%)	5 (9%)	0.0187

* One patient had missing data; ^±^ Calculated as CR + PR + SD; ^¶^ Calculated as CR + PR.

**Table 3:** Survival according to treatment.

Treatment Group	mOS (Months)	1-Year Survival Rate (%)	2-Year Survival Rate (%)	Survival at Data Closure (%)
Group A: all patients liver only metastases	16.5	56%	13%	39%
	16.1			
	19.0			
Group B: all patients liver only metastases	8.4	28%	5%	20%
	8.4			
	7.5			
Group C: all patients liver only metastases	10.5	37%	6%	19%
	10.5			
	10.5			
All patients	11.0	38%	7%	24%

**Table 4:** Multivariate analysis of predictors for survival.

Predictor	HR	95% CI	*p*-Value
Sorafenib/TACE	0.34	0.23–0.53	**<0.001**
Child–Pugh score	0.48	0.31–0.71	**<0.001**
ECOG PS	0.56	0.37–0.83	**0.002**
AFP ≥ 400 ng/mL	1.79	1.25–2.5	**0.006**
CLIP score 1–2	0.91	0.56–1.42	0.68
Zhao risk score < 11.5	0.87	0.56–1.42	0.53

Bold to illustrate significant *p* values.

**Table 5:** Sorafenib-related adverse events.

Adverse events	Sorafenib/TACE (Group A) *n* = 50	Sorafenib (Group B) *n* = 78
Any adverse event	86%	80%
Adverse events ≥ grade 3	34%	32%
Sorafenib dose reduction	66%	60%
Diarrhea	26%	22%
Hand–foot skin reaction	24%	17%
Fatigue	6%	8%
Sorafenib interruption	38%	35%
Hand–foot skin reaction	12%	10%
Diarrhea	10%	10%
Fatigue	6%	5%
Termination of sorafenib	12%	15%
Hand–foot skin reaction	4%	4%
Diarrhea	2%	3%
Fatigue	2%	3%

**Table 6:** Adverse events related to TACE occurring in ≥10% of the patients.

Adverse Events, n (%)	Sorafenib + TACE (*n* = 37)	TACE (*n* = 38)
Postembolization syndrome *	11 (30%)	13 (34%)
Abdominal pain	14 (41%)	17 (45%)
Nausea	12 (33%)	13 (34%)
Fever in absence of infection	10 (27%)	10 (26%)
Vomiting	5 (14%)	6 (16%)
New ascites	9 (24%)	8 (21%)
Fatigue	8 (22%)	5 (13%)
Liver dysfunction	6 (16%)	4 (11%)
Anorexia	5 (14%)	4 (11%)

* Postembolization syndrome did not require prolonged hospitalization (beyond 24 h) for post-treatment observation.

## Data Availability

The data presented in this study are available on request from the corresponding author. The data are not publicly available due to ethical restrictions.

## Supplementary Materials

The following are available online at https://www.mdpi.com/article/10.3390/cancers13092121/s1, File S1: Inclusion criteria and exclusion criteria.

## Abbreviations

HCC, hepatocellular carcinoma; BCLC, Barcelona Clinic Liver Cancer; DCR, disease control rate, SOC, standard of care; TACE, transarterial chemoembolization; PVI, portal vein infiltration; EHM, extrahepatic tumor manifestation; ECOG, Eastern Cooperative Group; SOC, standard of care; VEGFR, vascular growth factor receptor; CR, complete response; ORR; overall response rate; OS, overall survival; PFS, progression free survival; PR, partial remission; SD, stable disease; PD, progressive disease.
