# Supplementary material for: Combination of Sorafenib and Transarterial Chemoembolization in Selected Patients with Advanced-Stage Hepatocellular Carcinoma: A Retrospective Cohort Study at Three German Liver Centers
Source: Cancers (Basel). 2021 Apr 28;13(9):2121. doi: 10.3390/cancers13092121 (PMC8124282; doi:10.3390/cancers13092121)
Supplement: Supplementary file 1 [file cancers-13-02121-s001.zip › cancers-1159129-supplementary.pdf]

Inclusion criteria:

1. Hepatocellular carcinoma, diagnosed according to EASL guideline
   a. stage BCLC C
   b. Child-Pugh ≤9
   c. CLIP score < 4
   d. ECOG performance status ≤2
2. Not amenable to surgery
3. Age 18 years or older
4. At least one target lesion as defined by RECIST 1.1 criteria
5. TACE technically feasible
6. Sufficient renal function (creatinine <2,0 mg/dl)
7. Treatment and follow-up performed in one of the three participating centres (Frankfurt, Berlin, Munich; all in Germany)
8. For groups A and B: treatment with sorafenib for at least 30 days
9. Time period between initiation of sorafenib treatment and TACE no longer than 60 days
10. Prior treatment with sorafenib was allowed if the treatment time did not exceed three months

Exclusion criteria:

1. Portal vein thrombosis of the main portal branch
2. Other malignancies than HCC
3. Non-compliance
4. Patient amenable to curative treatment (such as surgery or transplantation) after sorafenib and/or TACE treatment
5. Progression to BCLC stage C during TACE treatment
